# Computed tomographic evaluation of cleft palate in one-day-old puppies

**DOI:** 10.1186/s12917-018-1642-6

**Published:** 2018-10-20

**Authors:** Filip Pankowski, Sławomir Paśko, Andrzej Max, Bartłomiej Szal, Małgorzata Dzierzęcka, Joanna Gruszczyńska, Paweł Szaro, Marek Gołębiowski, Bartłomiej Jan Bartyzel

**Affiliations:** 1 Department of Morphological Sciences, Faculty of Veterinary Medicine, Warsaw University of Life Sciences – SGGW, Warsaw, Poland; 2 Virtual Reality Techniques Division, Institute of Micromechanics and Photonics, Faculty of Mechatronics, Warsaw University of Technology, Warsaw, Poland; 3 Department of Small Animal Diseases with Clinic, Faculty of Veterinary Medicine, Warsaw University of Life Sciences – SGGW, Warsaw, Poland; 4 Department of Genetics and Animal Breeding, Faculty of Animal Sciences, Warsaw University of Life Sciences – SGGW, Warsaw, Poland; 5 1st Department of Radiology, Medical University of Warsaw, Warsaw, Poland

**Keywords:** 3D modeling, Anatomy, Dogs, Newborn, Radiology, STL

## Abstract

**Background:**

Cleft palate is a birth defect characterized by a lack of fusion between structures forming the palate. Causes include a multitude of factors, both genetic and environmental. Computed tomography (CT) is widely used to evaluate morphological features and diagnose head disorders in adult dogs. However, there is less data about its use in neonatal dogs. The purpose of this study was to perform CT evaluation of palatal defects in one-day-old puppies and to present a novel approach of 3D modeling in terms of cleft palate assessment.

**Results:**

Macroscopic and CT examinations were performed in 23 stillborn or euthanized purebred newborn puppies. On the basis of CT data, a 3D model was prepared and the cleft surface area was then calculated. A multi-stage approach, which utilised software such as 3D Slicer and Blender, was applied. Palatal defects were found in ten dogs, of which five had cleft palate, three had bilateral cleft lip and palate, one had a unilateral cleft lip and palate and one had a unilateral cleft lip. The surface area of the clefts ranged from 31 to 213 mm^2^, which made up respectfully 11 to 63% of the total surface area of the palate. No abnormalities were found in thirteen dogs and they made up the control group.

**Conclusions:**

Computed tomography and 3D modeling were very effective in evaluation of palatal disorders in newborn dogs. 3D models adapted to the natural curvature of the palate were created and more precise data was obtained. Morphological characteristics, CT findings and advanced image analysis of cleft palate in neonates obtained from these models increase the knowledge about this malformation in dogs.

**Electronic supplementary material:**

The online version of this article (10.1186/s12917-018-1642-6) contains supplementary material, which is available to authorized users.

## Background

Cleft palate, or palatoschisis, is a congenital defect characterized by a lack of fusion between structures forming the palate, resulting in a slit-shaped connection between the oral and nasal cavities [1]. There are many ways to classify oral cleft defects, one of which uses the embryological system classification commonly used for research and clinical purposes. In this developmental system there is a division into a primary and secondary palatal defects, depending on the time when the defect forms. Another approach is to classify the defect based on the anatomical structures affected. The primary palate is defined as the combined alveoli, incisive bones and upper lip. The secondary palate is defined as the combined soft and hard palates [2]. The hard palate is composed of palatine processes of the incisive bones, the palatine processes of the maxillae, and the horizontal plates of the palatine bones. These bony structures together form an osseous component, which is surrounded by soft tissue. Congenital clefts result from disrupted closure of the primary palate, secondary palate, or both concurrently. We can therefore simply classify the defect as cleft lip (CL), cleft palate (CP) or cleft lip and palate (CLP) depending on the part affected, i.e. primary palate, secondary palate, or both, respectively. These defects can occur unilaterally or bilaterally with variable degrees of severity and have many different morphological characteristics [2–4]. Categorization of the cleft can sometimes be problematic because of the lack of uniformity in the classification systems, therefore a new numerical system has been recently proposed. It is based on the LAHS scheme, which assesses four topographic areas (Lip, Alveolus, Hard and Soft palate) and is simple to use in everyday clinical practice. This classification is intended to facilitate reporting of cleft occurrence in dogs, and thus the exchange and storage of information [5].

The critical period for cleft palate formation in dogs is the 25th to 28th day of pregnancy [6]. Causes of congenital palatal defects are not fully known. They include a multitude of factors, both genetic and environmental, such as folic acid deficiency, mechanical injuries, and infectious causes [7, 8]. Animals with congenital defects are usually not bred, in order to eliminate the transmission of defects to their offspring. This elimination process may however prove insufficient, because the animals do not routinely undergo genetic tests and are excluded from breeding solely on the basis of their phenotype. Although cleft palate is a relatively common defect, data on its prevalence in the canine population is limited. Neonates are euthanized or die in the first days of life; this is often not reported by breeders. One study showed that the defect was observed in 3.4% of a total of 526 puppies and in 12.5% of 112 litters studied [9]. It was also found that brachycephalic breeds are predisposed to this defect [7]. An analysis of 12 litters comprising 52 puppies showed the defect in almost 27% of the dogs in a large Brittany spaniel colony [10]. In another study on a population of 2104 Pyrenean Shepherds, lip or palate clefts were observed in 47 out of 163 puppies from 37 litters [11]. The abnormality was also reported as one of the malformations found in one cloned German shepherd dog [12].

In most cases, clefts of a secondary palate results in death of the newborn puppy, since it is not able to suckle and ingest properly. Nasopharyngeal infections, middle ear diseases and aspiration pneumonia can also occur [13, 14]. Even though it is possible to diagnose cleft palate by means of a clinical examination, it may require additional tests to diagnose concomitant disorders and their severity. A diagnosis of the defect can be made by performing ultrasonography in utero in humans [15], but there are no reports of prenatal diagnosis of this malformation in veterinary medicine.

Computed tomography (CT) is an excellent modality for morphological evaluation of the head in adult dogs [2, 16]. However, there is less data about its use in newborn puppies. To the best of our knowledge, there are currently no studies on CT diagnosis of craniomaxillofacial abnormalities, especially the cleft palate, in one-day-old puppies. The purpose of this study was to evaluate congenital palatal abnormalities in neonate dogs. Particular attention was given to determine the cleft surface area on the basis of CT data. To do so, 3D modeling was used for virtual filling of the palatal defects. Measurement of the cleft surface in a simple CT reconstruction does not fully reflect this parameter due to the curvature of the palate surface. Direct determination of the cleft surface area using standard CT software is therefore not entirely precise.

## Results

Four each of Yorkshire Terriers, Cavalier King Charles Spaniels, English Bulldogs, two each of Labrador Retrievers and German Shepherds and one each of Chihuahua, Bullmastiff, French Mastiff, Staffordshire Bull Terrier, Golden Retriever, West Highland White Terrier and Basset Hound were included in the study.

**Fig. 1:**
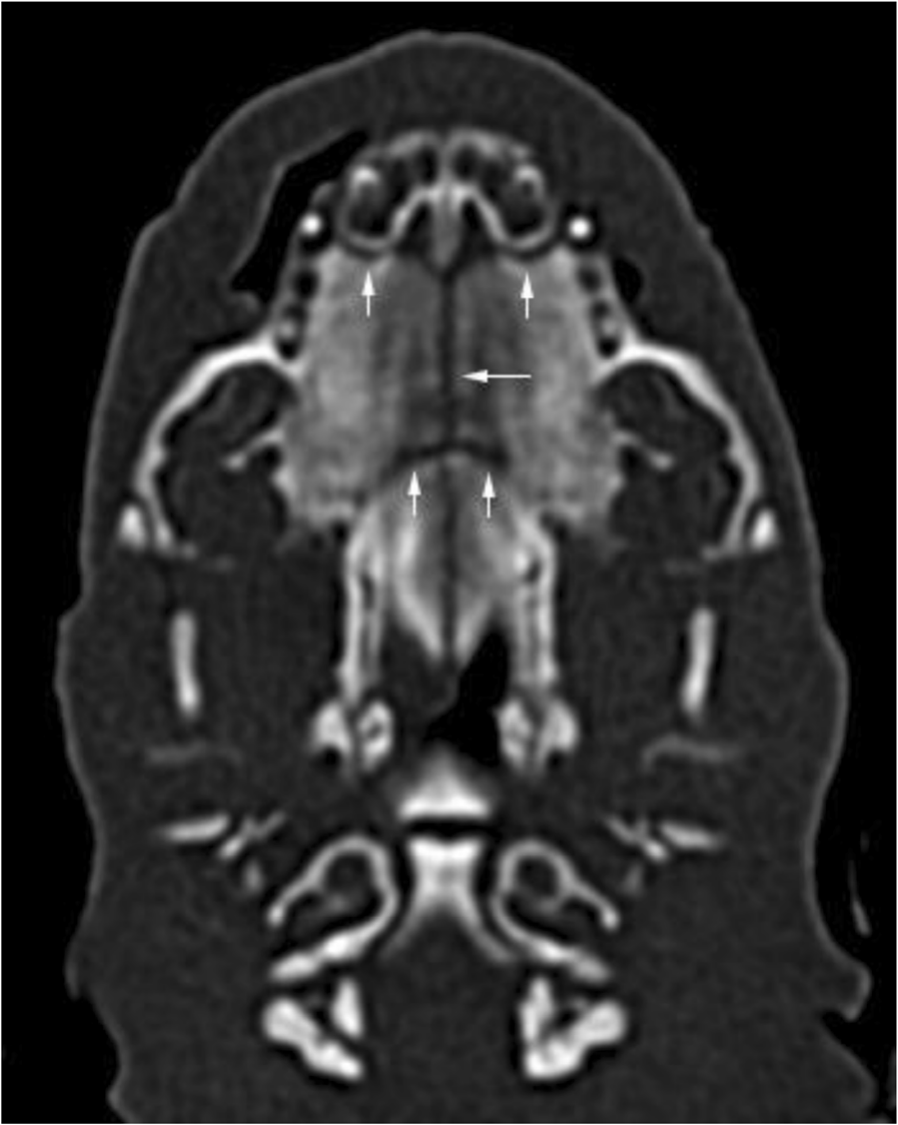
Radiolucent spaces between skull bones in a newborn dog without palatal abnormalities. Median palatine, palatomaxillary and incisivomaxillary sutures are visible (arrows). This is a normal finding and should not be confused with cleft palate. Level = 300 HU, width = 1500 HU

Normal development of the palate was visible in thirteen animals. On CT scans, there were no defects detected between the left and right side of the palate and the structures of the palate were fully developed, symmetrical and filled the entire surface of the palate. Radiolucent spaces reflecting non-mineralized sutures between bones forming the palate were present (Fig. 1). The median palatine suture was seen as a radiolucent fissure in a median plane between the left and right side of the palate. Palatomaxillary, incisivomaxillary and vomeroincisive sutures were also visible. Symmetrical palatine fissures were present in the rostral part of the palate.

**Fig. 2:**
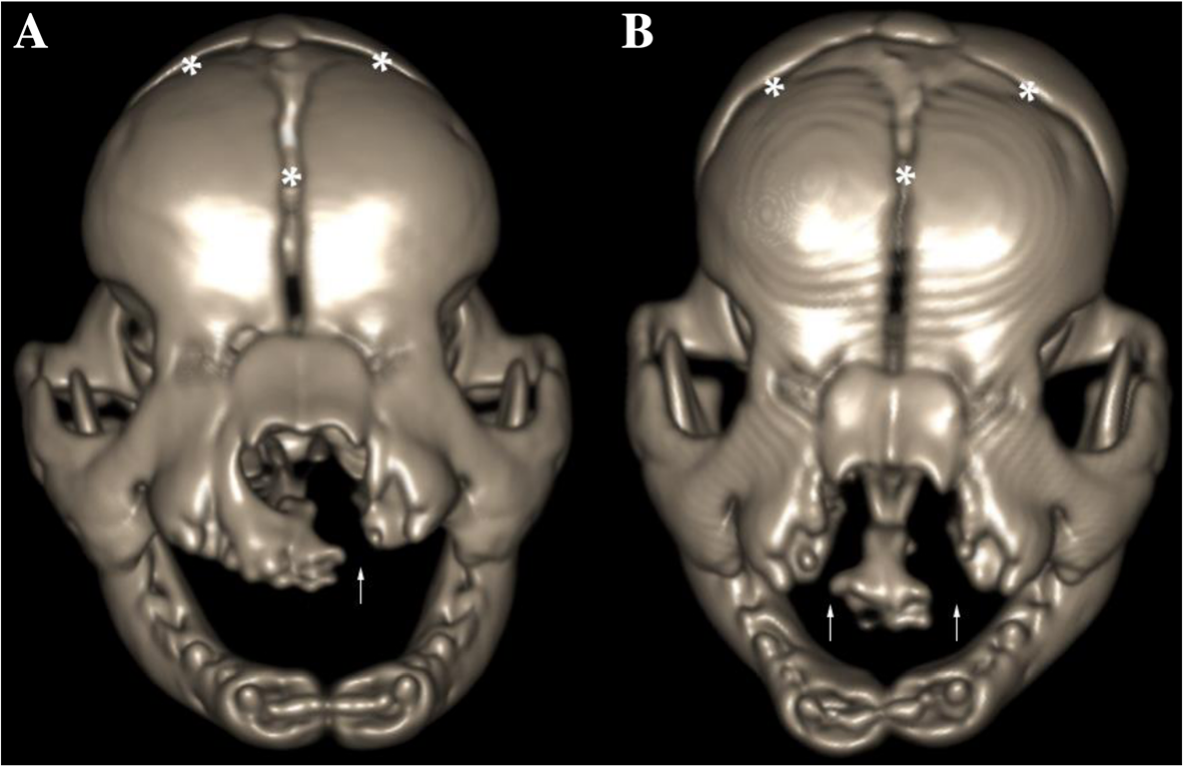
**a** Three-dimensional VR showing a unilateral cleft lip and cleft palate in an English Bulldog (arrow). **b** Bilateral cleft lip and cleft palate (arrows) in another English Bulldog. Notice areas corresponding to skull suture lines (*)

**Fig. 3:**
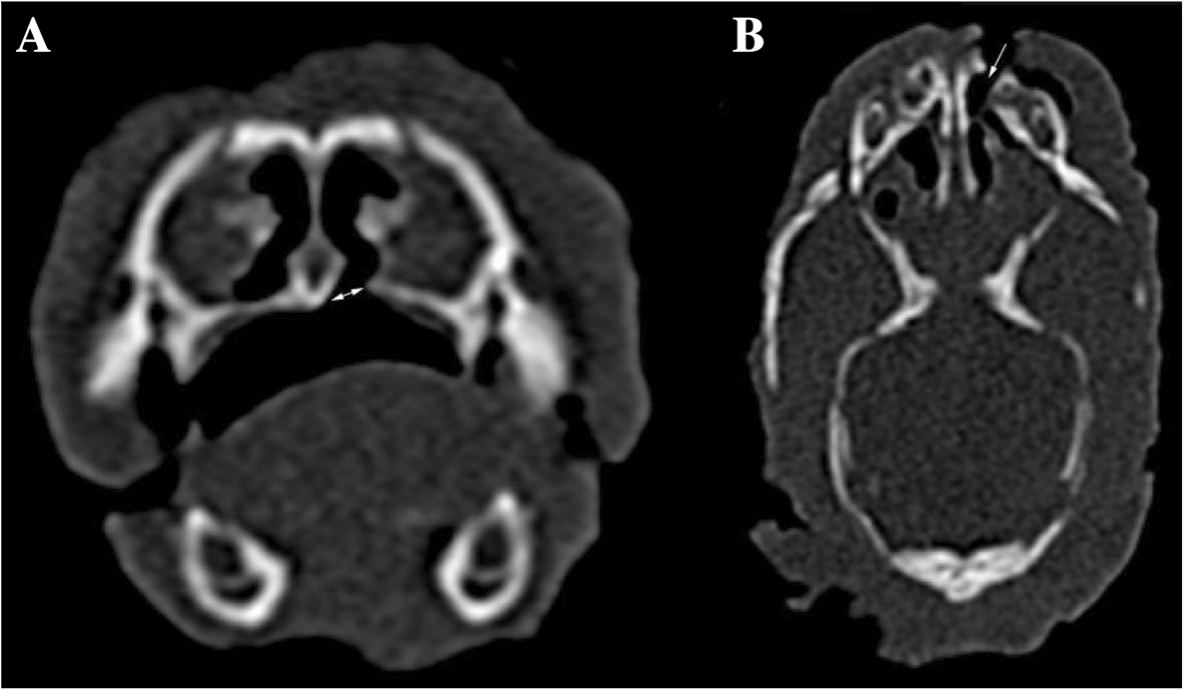
**a** Transverse CT image of cleft palate (arrow) in an English Bulldog. **b** Dorsal CT reconstruction of a unilateral cleft lip and cleft palate (arrow) in an English Bulldog. Level = 300 HU, width = 1500 HU

**Fig. 4:**
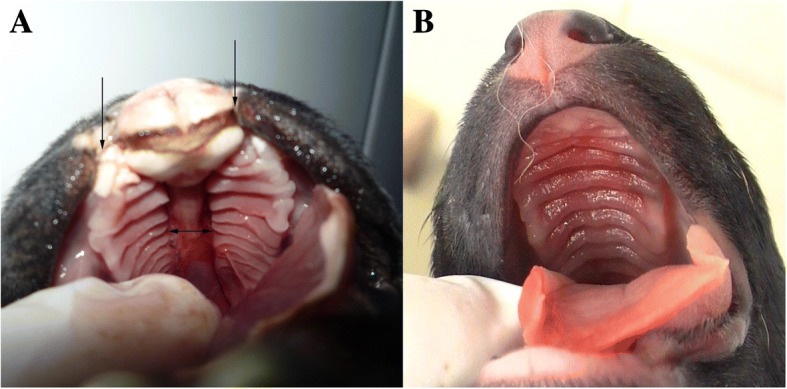
**a** Photograph of a bilateral cleft lip and cleft palate (arrows) in an English Bulldog. **b** Normal palate in a German Shepherd

Cleft of a secondary palate (i.e. CP) was the only defect in one of each of the Yorkshire Terrier, Golden Retriever, West Highland White Terrier, Basset Hound and English Bulldog. Bilateral clefts of the primary palate together with a cleft of a secondary palate (i.e. CLP) were present in a German Shepherd and two English Bulldogs (Fig. 2). An additional movie file shows this from different angles (Additional file 1). There was a unilateral cleft of the primary palate together with a cleft of the secondary palate (i.e. CLP) in another English Bulldog (Fig. 3). A unilateral cleft of the primary palate (i.e. CL) was present in the French Mastiff. Clefts of a primary palate were always accompanied by a discontinuity in the upper lip, which caused considerable shortening of the upper lip frenulum. Those clefts started at the gum ridge between bulges corresponding to unerupted 3rd incisors and canine teeth and extended up to the 9th palatal rugae. Communication between oral and nasal cavities was clearly visible (Fig. 4). Palpation revealed defects of the dorsolateral and ventrolateral nasal cartilages in all affected animals. The extent of the soft tissue clefts was smaller than the extent of osseous clefts in every case. The width of the clefts varied significantly between individuals, where the narrowest fissure was 2.1 mm wide in a transverse plane and the largest was 9.6 mm wide. 3D modeling allowed for a perfect reflection of the surface of the cleft palate considering the natural curvature of the surrounding structures. The surface area of the secondary cleft palates ranged from 31 to 213 mm^2^ which reflects 11 to 64% of the total surface area of the hard palate respectively (Table 1). A numerical classification code for every cleft is shown in Table 2.

**Table 1:** Surface area of the cleft of the secondary palate and its percentage of the total surface area of the hard palate

Dog No.	Breed	Total area of the hard palate (mm^2^)	Surface area of the cleft (mm^2^)	Percentage of the cleft in relation to total area of the hard palate
1	French Mastiff	572	62	11%
2	Golden Retriever	297	101	34%
3	West Highland White Terrier	142	79	56%
4	English Bulldog	356	121	34%
5	Basset Hound	313	188	60%
6	Yorkshire Terrier	97	45	46%
7	English Bulldog	352	113	32%
8	German Shepherd	153	31	20%
9	English Bulldog	351	168	48%
10	English Bulldog	331	213	64%

**Table 2:** Clefts classification according to the numerical classification system [5]

Cleft type	Dog No.	Breed	Diagnostic code
CL	1	French Mastiff	03–01–00-00
CP	2	Golden Retriever	00–00–33-33
	3	West Highland White Terrier	00–00–33-33
	4	English Bulldog	00–00–33-33
	5	Basset Hound	00–00–3-3
	6	Yorkshire Terrier	00–00–33-33
CLP	7	English Bulldog	03–03–03-03
	8	German Shepherd	22–33–33-33
	9	English Bulldog	33–33–33-33
	10	English Bulldog	33–33–33-33

Sections represent a clinical topographic area (first – upper lip, second – primary palate, third – hard palate, fourth – soft palate). The first number from each pair represents the right side of the palate, and the second number - the left side. Midline clefts are identified by a single number instead of a pair. Numbers 0 to 3 represent the degree of the cleft; the higher the number the more severe the cleft

## Discussion

Orofacial clefts are of high clinical and scientific importance in a group of many lethal developmental defects in dogs. The scope of this malformation can be evaluated only partially by means of clinical examination, including oral and dental inspection [17]. A comprehensive diagnostic assessment using CT offers a chance to diagnose disorders within structures that cannot be evaluated optimally during clinical examination. This particularly applies to the extent of the osseous component clefts versus the soft tissue clefts. In a recent study of 9 adult dogs with congenital palatal defect, it was found on CT that every dog had at least one associated craniomaxillofacial anomaly. The most common abnormalities were hypoplastic tympanic bullae, hypoplastic nasal turbinates, absent or cleft vomer and incomplete cribriform plate, and all of these can be potentially clinically significant [16]. Therefore, it is important to search for other abnormalities when a cleft palate is diagnosed. The knowledge of the range of changes obtained by CT scanning has practical applications; if changes are severe and there are many concomitant craniomaxillofacial anomalies resulting in a poor prognosis, the decision to euthanize the patient may be better justified.

Interpretation of a head CT scan in a one-day-old puppy is challenging and may require more time in comparison to an adult patient. This is mainly because of small size, but also distinctive tissue density and presence of unfused sutures. Bone defects were best visualized in the bone window, regardless the cleft type. Upper lip clefts were better visible in the soft tissue window, but the bone window was sufficient to recognize a cleft as well. Bone window enabled much better view of unfused sutures than the soft tissue window. 3D models perfectly depicted bone defects, because every voxel having less than 140 HU was removed. The thirteen normal one-day-old pups were useful for reference purposes and it is advisable to have images of normal animals for comparison of the observed anatomical structures. There are plenty of excellent sources depicting the radiological anatomy of adult dogs. However, to our knowledge, there is lack of similar data for newborn puppies. Such a source should be created in the future and CT can assist greatly in this. This type of data may be useful for radiologists that interpret images of animals at this age or veterinary forensic medicine professionals performing post-mortem computed tomography (PMCT) in order to assess the cause of death of a newborn. Additionally, access to stillborn or euthanized neonates is limited due to the fact that breeders are usually reluctant to reveal information about problems in their litter.

Radiolucent spaces between skull bones, including those forming the palate, should not be confused with defects such as cleft palate or fractures. The median palatine suture mineralizes with age and hence the radiolucent line is no longer observed in routine CT examinations of adult patients. Presence of unfused incisivomaxillary and vomeroincisive sutures in adult dogs with CL or CLP would be an abnormal finding contributing to the incisive bone instability [2]. A combination of digital modeling with data generated by CT can be helpful in assessing morphology of connections of the skull bones in the newborn [18]. The possibility of visualizing smaller defects in the soft tissue than defects of underlying osseous component denotes that CT is an excellent modality to show the actual extent of the cleft. It is one of the reasons CT examination should be a part of every surgical planning for cleft correction.

Currently, there are no methods of correcting canine palatal defects in the neonatal period. Surgeries are only performed when dogs are a few weeks of age or after they stopped growing. Until then, supportive treatment is necessary. Feeding that prevents milk aspiration is crucial. From around the third week of life puppies can be given soft homemade food or ready-made paediatric canine food. Dry food may be used in four-week-old dogs [13]. The patient should be constantly observed during that period, which is challenging, but may have a satisfying outcome [19]. There have been attempts at starting early treatment with palatal prostheses made from thermoplastic material, but these are still at an experimental stage [20, 21]. In veterinary medicine, surgical reconstruction of the viscerocranium aims only to enable effective sucking, swallowing and breathing reflexes. In human medicine however, multiple surgical reconstructions as well as psychological support are needed to assist patients with cleft palates to function properly in society.

A limitation of the study was that surface area of the cleft was modeled and calculated only for the osseous component of the hard palate and not for the primary palate defects. This was because unequivocal anatomic reference could not be determined in the region of the lip and alveoli. However, clefts of the hard palate occur more frequently and are clinically more important in practice. Innovative application of 3DSlicer and Blender indicates that such type of software can be successfully used as an instrument for CT data processing. Surface area of the cleft was modeled manually for every animal, but attempts to create a software that does this automatically may be of benefit in future.

## Conclusions

The study presented the evaluation of the cleft palate morphology in one-day-old puppies using computed tomographic data. In addition to the classic features describing the type of the cleft, a new classification system was applied, and the surface area of the cleft was calculated using 3D modeling. 3D modeling complemented the CT examination in determining the cleft surface area. A 3D model adapted to the natural curvature of the palate was created and more precise data was obtained. Determination of the surface area of ​​the entire hard palate allowed for a percentage assessment of the degree of the cleft in individual puppies. This ranged from 11 to 64%.

The presented utilisation of CT in diagnosis of cleft palate in one-day-old puppies seems to be the first study of this type. Computed tomography itself or enriched by a novel application of advanced image analysis can be effectively used for recognition and assessment of head abnormalities in canine neonates. This applies, above all, to the cleft palate - one of the most important congenital malformation.

## Methods

### Animals

The study was performed on 23 one-day-old stillborn or euthanized purebred puppies. Thirteen normal puppies made up the control group. The cadavers were kept frozen at a temperature of − 18 degrees Celsius until the study began. The specimens were then thawed at room temperature and macroscopic evaluation of the palate was performed. Photographs were taken and reviewed to aid interpretation of CT images when considered pertinent. A numerical classification code was determined for every type of cleft according to the Moura and Pimpão template [5].

### CT imaging

A CT scan of each of the puppies’ head was performed. The animals were scanned in sternal recumbency using a 64-slice CT scanner (Aquilion 64, Toshiba America Medical Systems Inc., Tustin, USA) with the following settings: 120 kV, 200 mA, a rotation time of 0.5 s, pitch 0.641, slice thickness of 0.5 mm, reconstruction increment of 0.5 mm and sharp algorithm (for bones). The matrix size was composed of 512 rows, each having 512 pixels (512 × 512). Scans were analysed using the IntelliSpace Portal work station (Philips, Amsterdam, the Netherlands) with the use of multi-planar reformatting (MPR) and three-dimensional volume rendering (VR). Images were reviewed in a bone window (level = 300 HU, width = 1500 HU) and a soft tissue window (level = 40 HU, width = 350 HU) with the ability to adjust window width and level as desired. Systematic evaluation of the palate was performed and abnormalities were recorded. Bone and soft tissue defects at the area of the palate were determined, especially losses in the body of the incisive bones, between the incisive bones and the palatine processes of the maxilla up to the horizontal plate of the palatine bone. Concomitant loss of incisor buds, presence of an upper lip cleft and length and width of every defect were recorded. Data on whether the defect included the primary or secondary palate, which side was affected and to what extent was recorded.

### 3D modeling

**Fig. 5:**
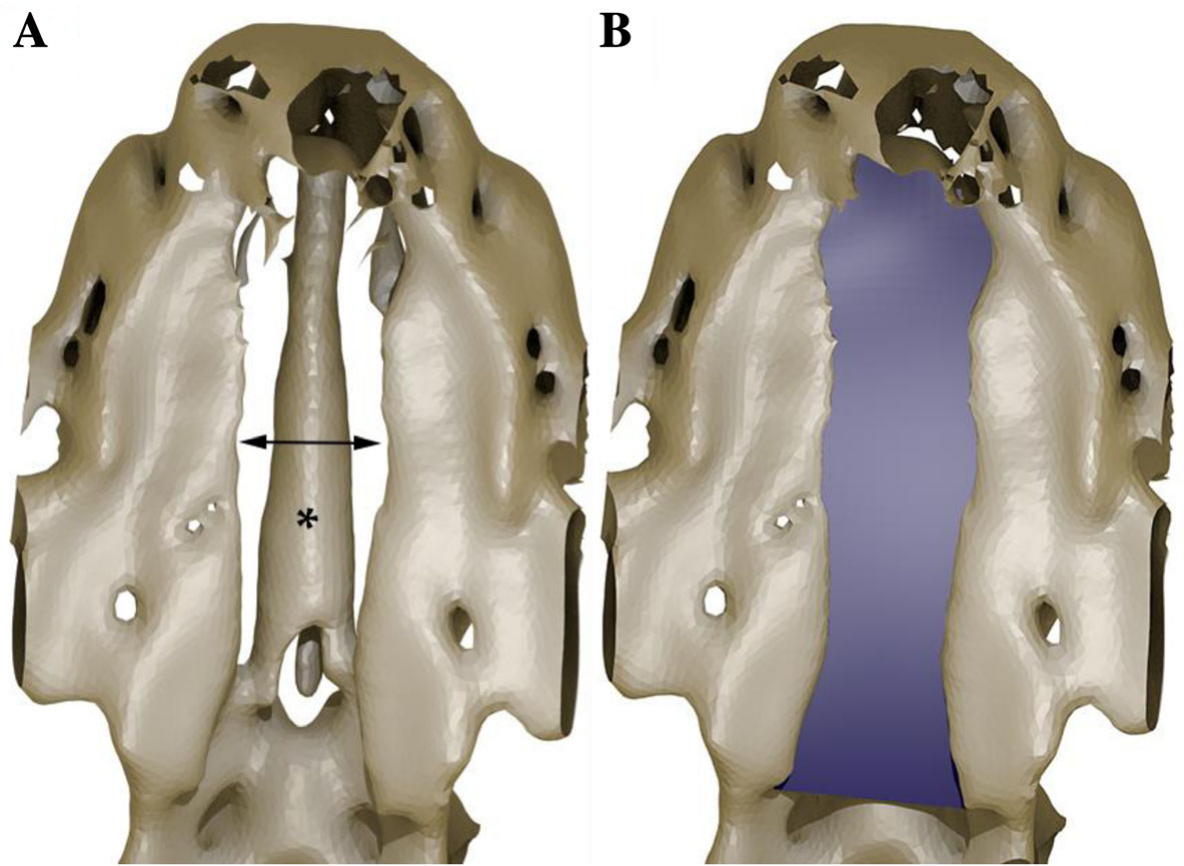
**a** Ventral view of an STL model showing a cleft of the hard palate (arrow) in an English bulldog. Notice the vomer situated above the cleft level (*). STL is a file format. **b** The same model as in (**a**) with filled surface area of the cleft

Length, width and surface area of the clefts of the secondary palate were measured. Calculation of the area was performed on a 3D model, which was obtained in a multi-stage operation. First, a three-dimensional STL model of a skull was generated in 3D Slicer (STL is a file format for 3D printing and 3D Slicer is a free, open-source platform for medical image analysis; http://www.slicer.org). The data was visualized in the volumetric form and the segmentation was performed using a function based on the cut-off threshold. A cut-off threshold of 140 Hounsfield units (HU) was used to distinguish bony structures from other tissues. The result of the segmentation was an unstructured triangulated surface, which was exported into STL format. Most of the triangles included in the model were then removed using Blender (free and open 3D creation software; Blender Foundation, http://www.blender.org), leaving only those that represented the analyzed area. Knife and Selection Tools were alternately used to indicate unnecessary areas. Uncluttered access was therefore obtained, and a virtual filling of the cleft area was performed by modeling with NURBS (Non-Uniform Rational B-Spline) surfaces (Fig. 5). The shape of the cleft surface was modified interactively by changing the location of knots. The operation was repeated until the best match to the existing structures of the palate was achieved. Next, the surface was changed to a polygon mesh, so that Knife and Selection Tools could be used to remove parts of the surface protruding beyond the outline of the defect. Finally, the surface area of the cleft of the secondary palate was calculated using a script in Python (programming language).

## Additional file

Additional file 1: Head of an English Bulldog with a bilateral cleft palate in three-dimensional VR shown from different angles. (MP4 243 kb)

## Abbreviations

CL: Cleft lip
CLP: Cleft lip and palate
CP: Cleft palate
CT: Computed tomography
HU: Hounsfield units
MPR: Multi-planar reformatting
STL: Stereolithography; standard triangle language; standard tessellation language
VR: Volume rendering
